# Distinguishing crystallographic from biological interfaces in protein complexes: role of intermolecular contacts and energetics for classification

**DOI:** 10.1186/s12859-018-2414-9

**Published:** 2018-11-30

**Authors:** Katarina Elez, Alexandre M. J. J. Bonvin, Anna Vangone

**Affiliations:** 1 Bijvoet Center for Biomolecular Research, Faculty of Science – Chemistry, Utrecht University, Padualaan 8, 3584 CH Utrecht, The Netherlands; 2 Present address: University of Bologna, Via Selmi 3, 40126 Bologna, Italy; 3 present address: Pharma Research and Early Development, Large Molecule Research, Roche Innovation Center Munich, Nonnenwald 2, Penzberg, Germany

**Keywords:** Biological interface, Crystal interfaces, Protein-protein interface, Classification, Intermolecular contacts, Residue contacts, Predictor, EPPIC, PISA

## Abstract

**Background:**

Study of macromolecular assemblies is fundamental to understand functions in cells. X-ray crystallography is the most common technique to solve their 3D structure at atomic resolution. In a crystal, however, both biologically-relevant interfaces and non-specific interfaces resulting from crystallographic packing are observed. Due to the complexity of the biological assemblies currently tackled, classifying those interfaces, i.e. distinguishing biological from crystal lattice interfaces, is not trivial and often prone to errors. In this context, analyzing the physico-chemical characteristics of biological/crystal interfaces can help researchers identify possible features that distinguish them and gain a better understanding of the systems.

**Results:**

In this work, we are providing new insights into the differences between biological and crystallographic complexes by focusing on “pair-properties” of interfaces that have not yet been fully investigated. We investigated properties such intermolecular residue-residue contacts (already successfully applied to the prediction of binding affinities) and interaction energies (electrostatic, Van der Waals and desolvation). By using the XtalMany and BioMany interface datasets, we show that interfacial residue contacts, classified as a function of their physico-chemical properties, can distinguish between biological and crystallographic interfaces. The energetic terms show, on average, higher values for crystal interfaces, reflecting a less stable interface due to crystal packing compared to biological interfaces. By using a variety of machine learning approaches, we trained a new interface classification predictor based on contacts and interaction energetic features. Our predictor reaches an accuracy in classifying biological vs crystal interfaces of 0.92, compared to 0.88 for EPPIC (one of the main state-of-the-art classifiers reporting same performance as PISA).

**Conclusion:**

In this work we have gained insights into the nature of intermolecular contacts and energetics terms distinguishing biological from crystallographic interfaces. Our findings might have a broader applicability in structural biology, for example for the identification of near native poses in docking. We implemented our classification approach into an easy-to-use and fast software, freely available to the scientific community from http://github.com/haddocking/interface-classifier.

**Electronic supplementary material:**

The online version of this article (10.1186/s12859-018-2414-9) contains supplementary material, which is available to authorized users.

## Background

Many essential cellular functions are mediated through specific protein-protein interactions. The physiological functions associated with those interactions are closely related to their three-dimensional (3D) structure. Hence, knowledge of the biologically-relevant 3D assembly of a complex is necessary for a proper understanding of interactions and their related function. Nowadays, structures of proteins and their assemblies are experimentally accessible through several techniques, the most common approach still being X-ray crystallography. Researchers are requested to deposit the coordinates of the solved crystal structures into the Protein Data Bank [1]. These, by convention, contain only the asymmetric unit (ASU). In the case of complexes, researchers are also asked to define the interface corresponding to what they believe to be the biologically-relevant assembly. This is however not trivial since protein crystal lattices contains usually various interfaces: The biologically-relevant one(s), occurring in solution or physiological state, and crystallographic ones, which are non-specific and artifacts of the crystallization process. Identification of biological interfaces cannot be inferred without ambiguity solely from crystallographic data. Additional, complementary experiments might have to be conducted, e.g. mutagenesis. Traditionally, the definition of the biological interface has often been performed by simple visual inspection since it has been shown that most crystallographic interfaces are smaller than the biological ones in terms of buried surface area [2–5]. However, the increasing complexity of biomolecular complexes solved nowadays has revealed biological and crystallographic interfaces of similar sizes, making their classification very challenging. For this reason, computational approaches have also been developed to tackle this problem.

Most of the computational classification methods proposed in the last decades rely on geometrical features [3, 6–8], evolution information [9–12], energetics aspects [13] or a combinations of those [14–17]. Among those, EPPIC [15] and PISA [13], both available as web servers, have shown the highest classification performance so far, with PISA being the current standard in the field. While PISA attempts to estimate the energy of binding, EPPIC relies on evolutionary and geometrical criteria.

The comparison of the physico-chemical characteristics of biological/crystal interfaces has helped researchers identify key features that distinguish the two. For example, the size of the interface, measured in terms of solvent-accessible area buried upon complex formation (BSA), has been shown, on average, to be larger in biological interfaces compared to crystallographic ones. A difference has also been found in the amino acids composition: Interfaces of biological complexes are enriched with aliphatic and aromatic residues, while the composition of crystallographic interfaces is not significantly different from that of the solvent accessible surface [3]. So far, no single complex property on its own has been shown to be specific enough to distinguish the two types of interfaces. Further analysis of properties of interfaces and the combination of the derived features are necessary to develop accurate classifiers and gain a better understanding of recognition mechanisms.

In this context, the role of pair-wise residue contacts (RCs) made at the interface has not yet been fully explored. The importance of intermolecular residue contacts at the interface of biological complexes has already been recognized and applied in several cases [18–21]: In docking, the number of native residue-residue contacts is used as one of the assessment criteria by the blind assessment experiment CAPRI [22], for scoring in various docking protocols [23–27], or for fast clustering [28, 29]. Recently, we have shown how the number and type of contacts can be a simple but robust predictor of binding affinity in protein-protein [30] and protein-ligand [31] complexes. Our predictor, implemented in the PRODIGY web-server [32, 33], is one the best performing so far reported in the literature over protein-protein complexes.

In this study, we analyze the properties of biological and crystallographic interfaces in terms of intermolecular residues contacts and interaction energies (such as electrostatic and van der Waals intermolecular energies and an empirically-derived desolvation energy term), in order to gain new insights into the different rules governing the two types of interfaces and better understand the interaction process. Based on our findings, we propose a simple, robust and competitive classification approach which is implemented into a program freely available at: http://github.com/haddocking/interface-classifier.

## Results

Given the importance of distinguishing a biologically-relevant interface from a crystallographic one when complementary experimental information is missing, we provide here new insights into the structural – focusing on the intermolecular residue contacts - and energetics differences between those interfaces, and present a novel approach to accurately classify them. For this purpose we use the Many datasets, compiled by Baskaran et al. (2014) [34]. It is the largest dataset reported so far in the literature, containing about 3000 biological (BioMany) and 3000 crystallographic (XtalMany) interfaces. From the original 5743 entries, we removed 8 cases that have too many clashes at the interface, making energetics calculations unreliable (see “Method” for details). For the remaining 5735 cases, we calculated contact-based structural properties, energetics values, and then applied machine learning approaches to train novel predictors for their classification into biological (bio) and crystallographic (xtal) interfaces. In the following we first present the differences of the various properties between the two sets of interfaces and then discuss the accuracy reached by the various classification approaches.

### Description of structural and energetic features

For each complex in the Many dataset, we first built any missing atoms (those not observed in the electron density) and then calculated the number and type of residue-residue contacts (RCs) made at the interfaces using a 5 Å cutoff (see also “Methods”). The 5 Å cutoff was the one giving the best prediction performance from a systematic scan between 3.5 Å and 12.5 Å (Additional file 1: Table S1). Considering that the interface buried surface area (BSA) is a widely-used property in the description of protein complexes and that it was originally considered an effective approach for such a classification, we included it in our analysis for comparison.

**Fig. 1:**
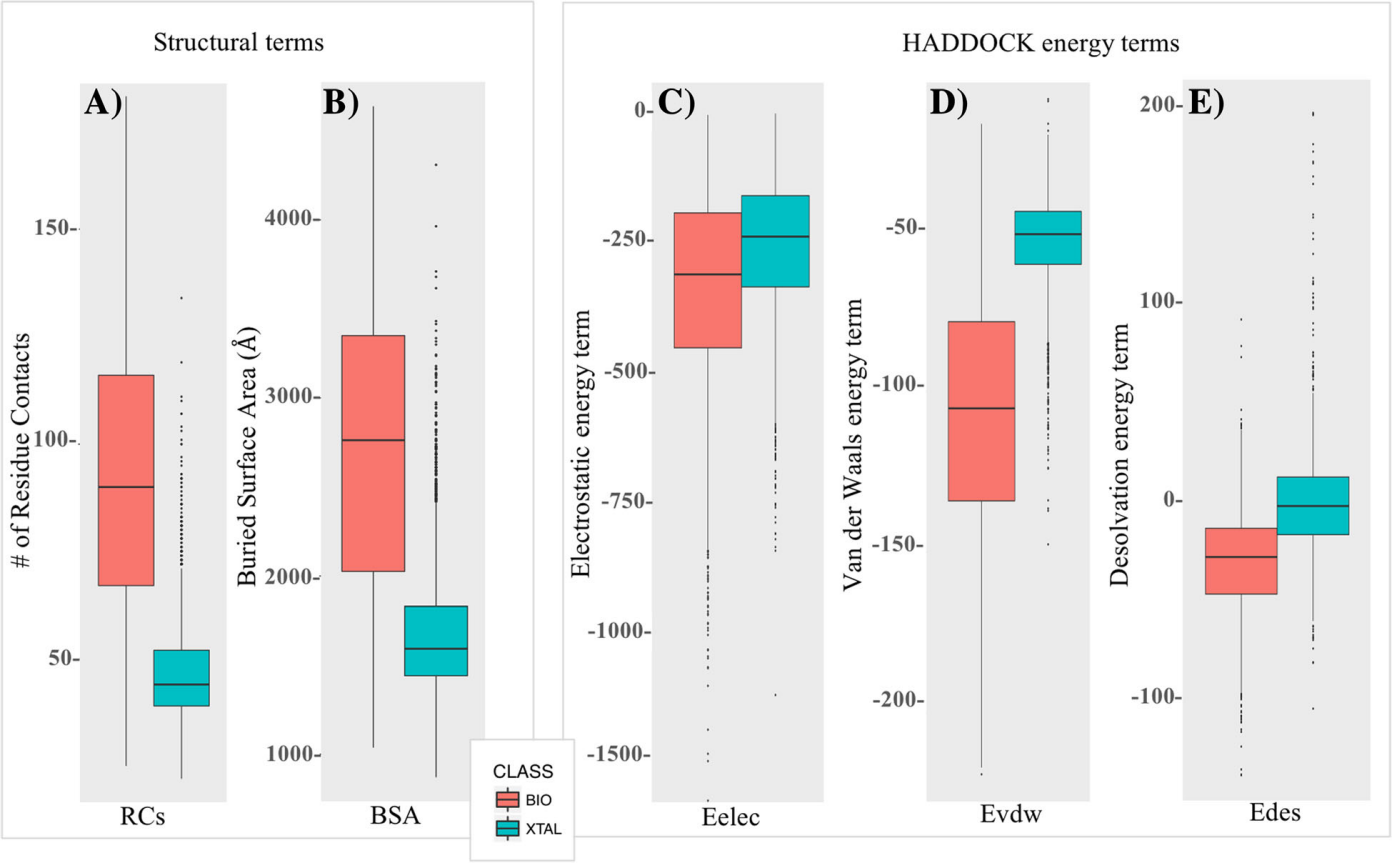
Boxplot of the structural and energetics properties. Boxplots of the interfacial residue-contacts (RCs) (*panel A*), the buried surface area (BSA) (*panel B*) and energetics values (*panels C, D, E*) are reported for the BioMany and XtalMany entries in pink and blue, respectively. The electrostatics (Eelec), Van der Waals (Evdw) and Desolvation (Edes) energies have been calculated with the HADDOCK refinement server. The black line in the middle of the boxes corresponds to the median, while the lower and upper hinges correspond to the 25th and 75th percentile, respectively, with the whiskers extending no longer than 1.5 times the interquartile range from the hinge. Point beyond the range are considered outliers and drawn as black points

In Fig. 1 we reported the distribution of RCs (*panel A*) and BSA (*panel B*) values for bio/xtal interfaces (in pink and blue, respectively). As expected, both in terms of BSA and RCs, crystallographic interfaces are generally smaller than biological ones, with averages values of 47 ± 11 and 1698 ± 359 Å^2^, for RCs and BSA, respectively, for crystallographic interfaces, versus 91 ± 31 and 2706 ± 803 Å^2^ for the biological interfaces. None of the RCs and BSA distributions is completely separated, which implies that they cannot be used as classification criteria by themselves. However, the residue contacts plots reveal better separated distributions with less outliers compared to BSA. This is in agreement with our previous observation that RCs are a better descriptor of binding affinity in protein-protein complexes than the BSA [30]. The number of RCs does not only reflect the size of the interface, as the BSA, but also its topology. For instance, by calculating the number of intermolecular residue contacts instead of the buried surface area, it is possible to capture differences between geometries that would allow different number of contacts despite similar buried surfaces. By evaluating the effect of the distance cut-off in defining a RC (range 3.5 Å - 12.5 Å) we observed the optimal cutoff at 5 Å, while the distributions trend is reproduced also at different cut-off values (see Additional file 1: Figure S1). In addition to the total number of residues RCs, we analyzed the density of contacts at the interface by calculating the link density (LD) (see Eq. 1 in Methods). It varies between 0 and 1, with higher values indicating a denser contact network at the interface. The LD, however, does not have a better discrimination power than the number of RCs (data not shown).

**Fig. 2:**
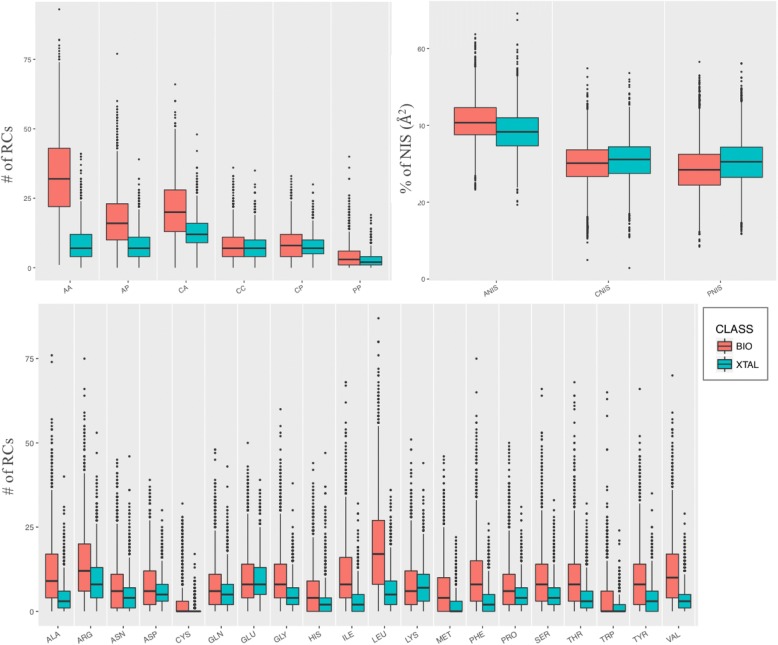
Boxplot of the structural properties divided by physico-chemical properties. Boxplots of the interfacial Residue-Contacts (RCs) and of the Non-Interacting Surface (NIS) classified by the charged/polar/apolar character of the residues (*top left panel and top right panel,* respectively). The analysis of the number of RCs for each of the 20 standard amino acids is reported in the bottom panel. BioMany and XtalMany entries are reported in pink and blue, respectively. The black line in the middle of the boxes corresponds to the median, while the lower and upper hinges correspond to the 25th and 75th percentile, respectively, with the whiskers extending no longer than 1.5 times the interquartile range from the hinge. Point beyond the range are considered outliers and drawn as black points

We then went into more details by classifying the RCs according to the interacting amino acid type (with a total of 20 RCs-classes, one for each standard amino acid) and their polar/apolar/charged character, with a total of 6 contact classes: charged-charged (CC), charged-polar (CP), charged-apolar (CA), polar-polar (PP), polar-apolar (PA) and apolar-apolar (AA). As we can observe in Fig. 2 (*top-left* panel), the largest difference between bio and xtal interfaces contacts is clearly found in the number of apolar-apolar contacts, while the smallest is in the number of polar-polar contacts. Biological interfaces are clearly richer in apolar-apolar contacts than crystallographic ones. This is in agreement with previous findings [35, 36] showing that the association of soluble proteins into larger complexes involves often hydrophobic patches, for which exposure to the solvent would be destabilizing. Crystallographic interfaces instead, which do not occur in solution, show no preference into the composition of the interfacial amino acids. This difference is clearly captured by AA-RCs distributions between bio and xtal interfaces. By looking at the specific amino acids contributions (Fig. 2, *bottom* panel), aliphatic apolar residues as LEU, ILE, VAL and ALA are the ones showing the largest differences in their number of contacts between bio and xtal interfaces.

We further analyzed the Non-Interacting Surface (NIS) properties (see “Methods”), since these have been described in previous works to contribute to the binding affinity in protein complexes [37, 38]. The percentages of polar (PNIS), apolar (ANIS) and charged (CNIS) residues on the non-interacting surface, does not reveal any significant differences between biological and crystallographic interfaces (Fig. 2, *top right* panel). The physico-chemical properties of the non-interacting surface are thus quite similar in both types of complexes.

Finally, in order to calculate the intermolecular Electrostatic (Eelec), Van der Waal (Evdw) and Desolvation (Edes) energies, we refined all interfaces of the Many dataset using the online HADDOCK refinement server [39] (see also “Method” for details). The various energy terms all point to more stable biological interfaces compared to the crystallographic ones, with lower energy values in all cases (Fig. 1, panels C, D and E). The intermolecular van der Waals energy seems the most discriminating energetic component between biological and crystallographic interfaces. It shows quite favorable (negative) values for biological interfaces with better separated distributions. Empirical desolvation energies show slightly overlapping distribution. The electrostatic energies, instead, are rather similar for both kind of interfaces.

### Machine learning training and testing of classification models

In order to distinguish between crystallographic and biological interfaces, we trained various predictors using different classification methods based on combinations of the above described properties. The best performing one was implemented into an easy-to-use software. For the purpose of training, the Many data set provides a broad and balanced data set. Ten fold cross-validation was performed by randomly dividing the data into ten subsets, 9/10 of whose used for training and 1/10 for testing. This process was repeated 10 times to reduce the randomness of the data partitioning. Five different machine learning algorithms were used to train the models: Bagging, Random Forest, Adaptive Boosting, Gradient Boosting and Neural Network. Their performance measured on the left-out testing data is reported in terms of accuracy (i.e. the percentage of entries correctly predicted, see Eq. 2 Methods).

Classification models were trained using nine different sets of features consisting of a combination of Structural properties (feature sets labelled as S1, S2, S3, S4, S5, S6), Energetics (sets E1 and E2) and a Combination on the two (set C). Details of the various features sets are reported in Table 1 (columns 1 and 2).

**Table 1:** Performance of classification models based on different features and training algorithms

	Training Features	Bagging	Random Forest	Adaptive Boosting	Gradient Boosting	Neural Network	Average
S1	BSA	0.74	0.74	0.81	0.81	0.55	0.73
		*(0.51)*	*(0.51)*	*(0.43)*	*(0.41)*	*(0.50)*	*(0.47)*
S2	RCs	0.86	0.86	0.85	0.86	0.85	0.86
		*(0.50)*	*(0.50)*	*(0.51)*	*(0.50)*	*(0.54)*	*(0.51)*
S3	CC, CP, CA, PP, AP, AA	0.89	0.90	0.89	0.89	0.89	0.89
		*(0.67)*	*(0.70)*	*(0.69)*	*(0.67)*	*(0.67)*	*(0.68)*
S4	CC, CP, CA, PP, AP, AA, ANIS, CNIS, PNIS	0.90 *(0.69)*	0.90 *(0.69)*	0.89 *(0.66)*	0.89 *(0.67)*	0.89 *(0.67)*	0.89 *(0.68)*
S5	CC, CP, CA, PP, AP, AA, LD, G, A, L, M, F, W, K, Q, E, S, P, V, I, C, Y, H, R, N, D, T	0.92 *(0.74)*	0.92 *(0.73)*	0.91 *(0.74)*	0.92 *(0.71)*	0.91 *(0.77)*	0.92 *(0.74)*
S6	CC, CP, CA, PP, AP, AA, ANIS, CNIS, PNIS, LD, G, A, L, M, F, W, K, Q, E, S, P, V, I, C, Y, H, R, N, D, T	0.92 *(0.73)*	0.92 *(0.75)*	0.91 *(0.74)*	0.93 *(0.70)*	0.92 *(0.76)*	0.92 *(0.74)*
E1	HS	0.76	0.76	0.83	0.82	0.82	0.80
		*(0.59)*	*(0.59)*	*(0.62)*	*(0.62)*	*(0.59)*	*(0.60)*
E2	Eelec, Evdw, Edes	0.87	0.87	0.87	0.87	0.85	0.87
		*(0.64)*	*(0.61)*	*(0.62)*	*(0.62)*	*(0.68)*	*(0.63)*
	CC, CP, CA, PP, AP, AA, ANIS, CNIS, PNIS, LD, G, A, L, M, F, W, K, Q, E, S, P, V, I, C, Y, H, R, N, D, T, Eelec, Evdw, Edes	0.92 *(0.72)*	0.93 *(0.73)*	0.92 *(0.74)*	0.93 *(0.72)*	0.90 *(0.77)*	0.92 *(0.74)*
C							

Accuracy values calculated according Eq. 2 in “Methods”

The predictive accuracies have been reported for several classification models tested. Nine sets of features have been used to train new predictive models, based on structural properties (S1, S2, S3, S4, S4, S6), energetics (E1, E2) and a combination of structure and energetics (C). For each set of training features, five machine learning algorithms have been used for the training (Bagging, Random Forest, Adaptive Boosting, Gradient Boosting and Neural Network). For the trained models, the accuracies on the Many [34] and the DC [15] (numbers in brackets) datasets are reported. The accuracy on the Many is reported as average of the 10-fold cross validation. In brackets the accuracy over the DC dataset is reported

**Fig. 3:**
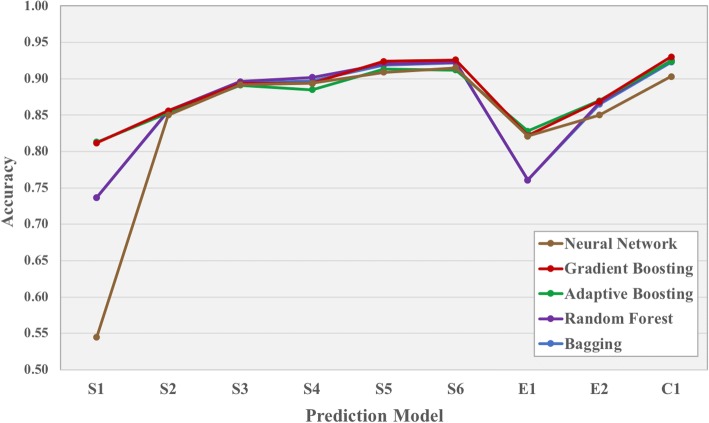
Accuracy of machine learning classifiers. Prediction accuracies (y-axis) of the various predictors as a function of the feature set used for training (x-axis). The training sets consist of structural properties (S1, S2, S3, S4, S4, S6), energetics (E1, E2) and a combination of both (C). Refer to Table 1 for the detailed list of features included in each set. Five different machine learning algorithms have been used for the training: Bagging, Random Forest, Adaptive Boosting, Gradient Boosting and Neural Network, reported in blue, purple, green, red and brown, respectively

From our analysis, models based on structural features (i.e., the ones trained on the S sets) achieve, on average, a higher accuracy that energy-based ones (E sets) (see Fig. 3 and Table 1). Interestingly, all five machine learning algorithms give rather consistent answers over the various feature sets (Fig. 3), which underscores the robustness of our training results. The BSA is the structural feature (set S1) that performs the worst, with an average accuracy of 0.73 ± 0.11 over the five different machine learning algorithms used. The combination of residue-contacts classified by amino acid type and physico-chemical properties (S5 set) gives the best classification performance, reaching an average accuracy of 0.92 ± 0.01 (set S5). The NIS properties do not add much to the trained models, which was to be expected considering their similarity between the bio/xtal interfaces: Models trained on the S4 and S6 sets show similar accuracy than the ones trained on S3 and S5 (the only difference between those being the addition of NIS properties in the training features). Overall, the best predictive model over the 5 algorithms is the one trained on both structural/energetics features together (set C). However, considering the computational time and resources needed to perform energetics calculations on protein complexes (i.e. running the HADDOCK refinement), the little gain in accuracy (average accuracy of 0.920 ± 0.01 vs 0.916 ± 0.01 of S5-models) can be ignored, making the simple and fast contact-based models best suited for classification purpose. We therefore decided to focus on the S5-set models. We used this set to analyze the importance of the various features. By applying a recursive feature elimination, we identify all the features (i.e., the one of set S5) as important (Additional files 1: Table S2). The most and least important feature for the classification is the number of contacts between two apolar residues at the interface (AA) and the number of contacts between two polar residues at the interface (PP), respectively. The last 5 ranked features in terms of importance (LYS, CC, TRP, ASN and PP) were not selected by the feature selection and hence excluded. On the remaining 22 we optimized (see Methods) the five machine learning algorithms, which were initially trained using default settings, to find the best predictive model to discriminate xtal from bio interfaces, the results of which are reported in Table 2. We finally selected as best classifier the Random Forest approach with 250 base estimators and the maximum number of features to consider when looking for the best split set to log2 on the total number of features. Its accuracy on the entire training dataset is 0.92.

**Table 2:** Optimization of the machine learning classifiers

	Classification accuracy on the MANY dataset	Classification accuracy on the independent DC dataset
Bagging	0.92	0.73
Random Forest	0.92	0.74
Adaptive Boosting	0.92	0.74
Gradient Boosting	0.93	0.74
Neural Network	0.91	0.75

The maximum accuracy reached by optimizing the settings is reported by each classifier for the Many (as average over the 10-fold cross-validation) and the DC datasets

### Validation on an independent dataset

To further assess the performance of our classifiers, we applied them to the independent DC dataset [15] that consists of about manually curated complexes with 80 xtal and 80 bio interfaces not present in the training dataset. The observed accuracies of the different models on the DC set are consistent with the training set ones, although showing lower values (Tables 1 and 2).

## Discussion

### Limit of the classifier and comparison with current state of art

Our final pair-wise contact-based model was trained over the S5 set of features (RCs divided by type/physico-chemical characteristics and Link Density), based on a Random Forest approach. On the Many dataset, our classifier shows a sensitivity and specificity of 91% and 93%, respectively; in other words, we correctly predict 91% and 93% of the biological and crystallographic interfaces, respectively, while we misinterpret about 7% of the xtal cases as bio, and 9% of the bio cases as xtal (average over the 10 test sets from the 10 fold training on the Many entries). We compared our results with the performance reported for the state of the art methods EPPIC [15] and PISA [13]. The concept at the basis of these prediction methods are different. PISA is a well-establish method in the field based on a thermodynamic estimation of the interface stability in order to predict whether it should exist in solution (biological interface) or not (crystallographic interface). EPPIC is a more recent method, based on evolutionary criteria; It uses a geometrical measure (number of buried residues) and two evolutionary indicators based on the sequence entropy of homolog sequences.

PISA and EPPIC have comparable performance. They have shown the same recall on a PDB-wide scale of 88% of PDB-interfaces [34]. Comparing our performance on the Many dataset, we reached a prediction accuracy of 92% versus the 88% of EPPIC (as reported in [34]). On the smaller DC dataset (about 160 cases vs the 5735 of the Many), our accuracy is lower with 74%, against 81% for EPPIC and 79% for PISA (data reported in [40]). However, the performance of EPPIC on the DC dataset has only been calculated for entries for which enough evolutionary information was found (about 82% of the DC entries). In fact, being an evolutionary-based method, its principal limitation is the sequence alignment depth. Next to its competitive performance, our predictor is very fast, returning predictions within a few seconds, compared to the minutes needed for the EPPIC and PISA web-servers. Our predictor is freely available for download from our GitHub repository (https://github.com/haddocking/interface-classifier).

## Conclusions

We have carried out the first study focused on using residue contacts and interaction energies to distinguish biological from crystallographic interfaces. The number and nature of residue and atom contacts have already been shown to be good descriptors of binding affinity in protein-protein [30] and protein-ligand [31] complexes. Here they have been shown to be good descriptors for the classification of interfaces in crystal structures, even when no other energetics contributions are included. This allowed us to train a simple machine learning predictor for fast and robust classification. Its simplicity, together with the advantage that energetic calculations are not required per se (which might depend on how complete the crystal structures are) nor are sequence alignments, should make it a useful tool for structural biology. We implemented our classification approach into an easy-to-use and fast software, freely available to the scientific community from http://github.com/haddocking/interface-classifier. We aim in a near future to offer it as a web server as an extension of our related binding affinity server PRODIGY [33]. Inter-residue contacts, which have now proven valuable features for both binding affinity prediction and crystallographic interface classification, have the potential to benefit other fields of research in structural biology, such the identification of near natives poses in docking.

## Methods

### The benchmark

In order to study and compare biological and crystallographic interfaces, we used the bio/xtal complexes in the “.pdb” format retrieved from the automatically generated Many dataset [34]. The 2831 biological entries (BioMany) includes dimeric crystal structures with interfaces present in multiple crystal forms retrieved from the ProtCID database [12, 41]. The 2913 crystallographic interfaces (XtalMany) are instead derived from interfaces that would lead to infinite assemblies (concept firstly introduced by Monod [42]). The interfaces span a difficult-to-classify interfacial area range of 500–2000 Å and represent a quite extensive and balanced set.

### Interface refinement and calculation of energetic features

Each PDB entry of the Many dataset was refined by using the refinement protocol of our HADDOCK web server [39, 43] (http://haddock.science.uu.nl/services/HADDOCK2.2/haddockserver-refinement.html). HADDOCK automatically builds eventually missing atoms and side-chains, which is necessary in order to get a more realistic model of the interaction, and refine the interface in explicit solvent using the TIP3P water model and the Optimized Potentials for Liquid Simulations (OPLS) force field [44] with an 8.5 Å cut-off for the calculations of the non-bonded interactions. The intermolecular energy terms and buried surface area (BSA) of the refined complexes were extracted from the top ranked HADDOCK model. The HADDOCK-derived features are:

- Evdw, the intermolecular van der Waals energy described by a 12–6 Lennard-Jones potential.
- Eelec, the intermolecular electrostatic energy described by a Coulomb potential.
- Edes, an empirical desolvation energy term [45].
- BSA, the buried surface area calculated by taking the difference between the sum of the solvent accessible surface area (SASA) for each individual protein and the SASA of the protein complex using 1.4 Å water probe radius.

We changed the non-standard amino acid MSE in MET in three cases: 5cnp_3, 3wnb_1 and 4py9_2. From the original dataset consisting of 5743 cases, we removed 8 entries that presented too many clashes at the interface, making the refinement unreliable: 1rzm_1, 3qv9_2, 2v9y_2, 4e0h_1, 3n94_1, 3axc_1, 4izv_2, 2bh7_2.

### Contact-based structural properties

Intermolecular contacts were calculated from the HADDOCK models with built missing side-chains, but before refinement. Contacts were obtained through our PRODIGY web server [33] (http://milou.science.uu.nl/services/PRODIGY/) and its stand-alone version available from GitHub (https://github.com/haddocking/prodigy). The following features were calculated:

- *Pair-wise Residue Contacts (RCs).* Two residues were considered in contact if any atom pair of the two residues is closer than a defined cut-off distance of 5.0 Å. To systematically evaluate the impact of the cut-off values on the results, we varied the cut-off from 3.5 Å to 12 Å. RCs were further classified by the types of the residues in contact: charged-charged (CC), charged-polar (CP), charged-apolar (CA), polar-polar (PP), polar-apolar (PA) and apolar-apolar (AA). Residue classes were the same as reported in Vangone and Bonvin 2015 [30]: N, Q, S and T as polar, E, D, H, K and R as charged and A, C, G, F, I, M, L, P, W, V and Y as apolar.
- *RCs per amino acid.* For each of the 20 standard amino acids, the number of RCs in which the amino acid is involved was calculated.
- *Link Density (LD)*, that expresses the density of the interfacial network. This is calculated as the total number of RCs at the interface divided by the maximum possible number of pair-wise contacts for that interface, as:

1 $$ LD=\frac{RCs}{Res1\ast Res2} $$

where Res1 and Res2 are the number of residues involved in RCs for interface 1 and 2, respectively.

As reported in [37, 38], the Non-Interacting Surface (NIS) is defined as the residues that show a maximum change of 5% between unbound and bound forms in terms of relative solvent accessibility. The percentage of polar, apolar and charged NIS (PNIS, ANIS and CNIS, respectively) are defined as the percentage the number of polar/apolar/charged residues divided by the total number of the residues on the NIS. Those properties have been obtained by the PRODIGY web server as well [33].

### Machine learning and assessment of the prediction

We used five machine learning algorithms from the scikit-learn python package (http://jmlr.csail.mit.edu/papers/v12/pedregosa11a.html) to train our classification models: Bagging, Random Forest, Adaptive Boosting, Gradient Boosting and Neural Network. We validated our results applying a 10-fold cross-validation approach on the training dataset. We systematically evaluated the impact of different settings of the machine learning algorithms on the accuracy. We tuned the hyper-parameters of the models by performing an exhaustive grid search on the selected parameters. We explored different values for the number of trees in the 4 ensemble methods (Bagging, Random Forest, Adaptive Boosting and Gradient Boosting). For Bagging and Random Forest, the maximum number of features to consider when looking for the best split was also changed. In addition, we optimized the learning rate for the two boosting methods (Adaptive Boosting and Gradient Boosting) and the maximum depth of the individual tree, in the case of the latter. The Neural Network was fine-tuned on the size of the hidden layer and its activation function.

A Random Forest of 500 trees was used to analyze the feature importance and perform recursive feature elimination with cross-validation in scikit-learn. First, the estimator is trained on the initial set of features and the importance of each feature is obtained through a feature importance attribute. Then, the least important features are pruned from the current set. The procedure is recursively repeated on the pruned set until there is no increase in the accuracy. The feature importance is measured by the decrease of Gini impurity index when splitting on a feature, averaged over all trees.

The prediction performance was evaluated by calculating the accuracy defined as:

2 $$ Accuracy=\frac{TP+ TN}{P+N} $$

where TP and TN are the true positive and true negative cases, respectively, and P and N are the total number of positive and negative cases, respectively.

### Data and model availability

All the HADDOCK-refined models are available on the SBGrid Data repository https://data.sbgrid.org/dataset/566/ [46]. The bio/xtal classification predictor and the features used for training and testing are freely available on GitHub: http://github.com/haddocking/interface-classifier.

## Additional file

Additional file 1: **Figure S1.** Boxplot of the RCs as function of the distance cut-off. **Table S1.** Evaluation of machine learning accuracy models VS distance cut-off. **Table S2.** Feature selection on the final predictive model. (ZIP 207 kb)

## Abbreviations

BSA: Buried Surface Area
EPPIC: Evolutionary Protein-Protein Interface Classifier
HADDOCK: High Ambiguity Driven biomolecular DOCKing
NIS: Non Interacting Surface
PDB: Protein Data Bank
PISA: Protein Interface, Surface and Assemblies
PRODIGY: PROtein binDIng enerGY prediction
RCs: Residue-residue Contacts
