# Supplementary material for: Distinguishing crystallographic from biological interfaces in protein complexes: role of intermolecular contacts and energetics for classification
Source: BMC Bioinformatics. 2018 Nov 30;19(Suppl 15):438. doi: 10.1186/s12859-018-2414-9 (PMC6266931; doi:10.1186/s12859-018-2414-9)
Supplement: Supplementary file 1 — Figure S1. Boxplot of the RCs as function of the distance cut-off. Table S1. Evaluation of machine learning accuracy models VS distance cut-off. Table S2. Feature selection on the final predictive model. (ZIP 207 kb) [file 12859_2018_2414_MOESM1_ESM.zip › S2-S1.docx]

**Distinguishing crystallographic from biological interfaces in protein complexes: Role of intermolecular contacts and energetics for classification**

Katarina Elez^1,2^, Alexandre M.J.J. Bonvin^1*^ and Anna Vangone^1,3*^

^1^Bijvoet Center for Biomolecular Research, Faculty of Science - Chemistry, Utrecht University, Padualaan 8, 3584CH, Utrecht, The Netherlands

^2^ Current address: Department of Biological, Geological and Environmental Sciences – BiGeA, University of Bologna, Via Selmi 3 40126, Bologna, Italy.

^3^ Current address: Pharma Research and Early Development, Large Molecule Research, Roche Innovation Center Munich, Nonnenwald 2 Penzberg, Germany.

*Correspondence: a.m.j.j.bonvin@uu.nl, a.vangone@gmail.com


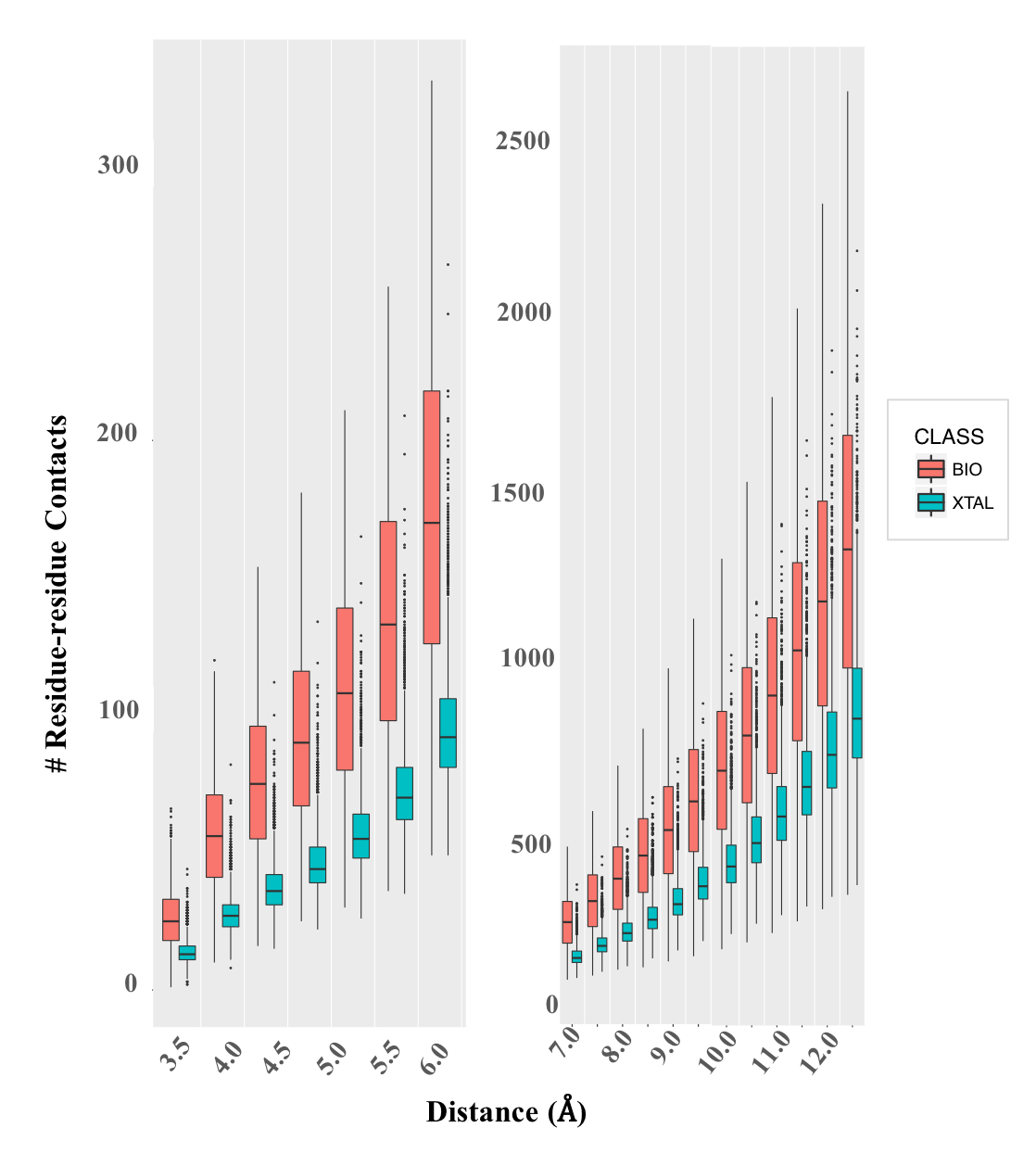


**Figure S1. Boxplot of the RCs as function of the distance cut-off .** Boxplots of the number of interfacial residue-contacts (RCs) as a function of the distance cut-off are reported for the BioMany and XtalMany entries in pink and blue, respectively. The black in the middle of the boxes corresponds to the median, while the lower and upper hinges correspond to the 25^th^ and 75^th^ percentile, respectively, with the whiskers extending no longer than 1.5 times the interquartile range from the hinge. Point beyond the range are considered outliers and drawn as black points.

**Table S1.** **Evaluation of machine learning accuracy models VS distance cut-off.** In the table, the accuracies of each machine learning predictor are reported (columns 2-6) per distance threshold (column 1) used to define distance-dependent properties. Average over the 5 predictors accuracies have been reported in column 7. Predictors have been trained on the distance-dependent properties: number of residue-residue contacts in charges/charged, charged/polar, charged/apolar, polar/polar, polar/apolar, apolar/apolar and link density. In bold is reported the cut-off value reaching the best average accuracy.

| **Cut-off** | **Bagging** | **Random Forest** | **Adaptive Boosting** | **Gradient Boosting** | **Neural Network** | **Average** |
| --- | --- | --- | --- | --- | --- | --- |
| 3.5 | 0.81 | 0.815 | 0.836 | 0.841 | 0.831 | 0.827 |
| 4.0 | 0.877 | 0.887 | 0.885 | 0.89 | 0.889 | 0.886 |
| 4.5 | 0.886 | 0.89 | 0.895 | 0.895 | 0.888 | 0.891 |
| **5.0** | **0.885** | **0.892** | **0.889** | **0.897** | **0.89** | **0.891** |
| 5.5 | 0.884 | 0.886 | 0.889 | 0.89 | 0.886 | 0.887 |
| 6.0 | 0.872 | 0.884 | 0.88 | 0.887 | 0.88 | 0.881 |
| 6.5 | 0.876 | 0.88 | 0.885 | 0.887 | 0.883 | 0.882 |
| 7.0 | 0.885 | 0.889 | 0.89 | 0.891 | 0.881 | 0.887 |
| 7.5 | 0.884 | 0.893 | 0.888 | 0.894 | 0.876 | 0.887 |
| 8.0 | 0.889 | 0.887 | 0.887 | 0.894 | 0.872 | 0.886 |
| 8.5 | 0.886 | 0.886 | 0.886 | 0.891 | 0.859 | 0.882 |
| 9.0 | 0.884 | 0.885 | 0.887 | 0.895 | 0.865 | 0.883 |
| 9.5 | 0.885 | 0.888 | 0.884 | 0.896 | 0.861 | 0.883 |
| 10.0 | 0.887 | 0.887 | 0.884 | 0.894 | 0.845 | 0.879 |
| 10.5 | 0.88 | 0.882 | 0.884 | 0.891 | 0.843 | 0.876 |
| 11.0 | 0.879 | 0.878 | 0.878 | 0.89 | 0.842 | 0.873 |
| 11.5 | 0.88 | 0.88 | 0.877 | 0.889 | 0.828 | 0.871 |
| 12.0 | 0.881 | 0.881 | 0.874 | 0.884 | 0.816 | 0.867 |
| 12.5 | 0.881 | 0.88 | 0.874 | 0.884 | 0.809 | 0.866 |

**Table S2.** **Feature selection on the final predictive model.** Features have been ranked according to the relative importance in the classifier, obtained through a feature importance attribute (the higher, the more important is the feature - for details, see “Methods”). The features reported in this table are the interfacial residue-contacts classified according to the amino acid involved (with a total of 20 RCs-classes, one for each standard amino acid, reported as single-code in the table) and their polar/apolar/charged character, with a total of 6 classes: charged-charged (CC), charged-polar (CP), charged-apolar (CA), polar-polar (PP), polar-apolar (PA) and apolar-apolar (AA) contacts.

| AA | 0.270 |
| --- | --- |
| LEU | 0.096 |
| VAL | 0.079 |
| AP | 0.065 |
| LD | 0.057 |
| ILE | 0.051 |
| PHE | 0.043 |
| CA | 0.042 |
| TYR | 0.035 |
| ALA | 0.022 |
| THR | 0.022 |
| HIS | 0.021 |
| MET | 0.016 |
| ASP | 0.016 |
| ARG | 0.015 |
| PRO | 0.015 |
| GLY | 0.015 |
| CP | 0.015 |
| CYS | 0.015 |
| SER | 0.014 |
| GLU | 0.014 |
| GLN | 0.012 |
| LYS | 0.012 |
| CC | 0.011 |
| TRP | 0.011 |
| ASN | 0.010 |
| PP | 0.009 |
