# Supplementary figures and images for: Distinguishing crystallographic from biological interfaces in protein complexes: role of intermolecular contacts and energetics for classification
Source: BMC Bioinformatics. 2018 Nov 30;19(Suppl 15):438. doi: 10.1186/s12859-018-2414-9 (PMC6266931; doi:10.1186/s12859-018-2414-9)

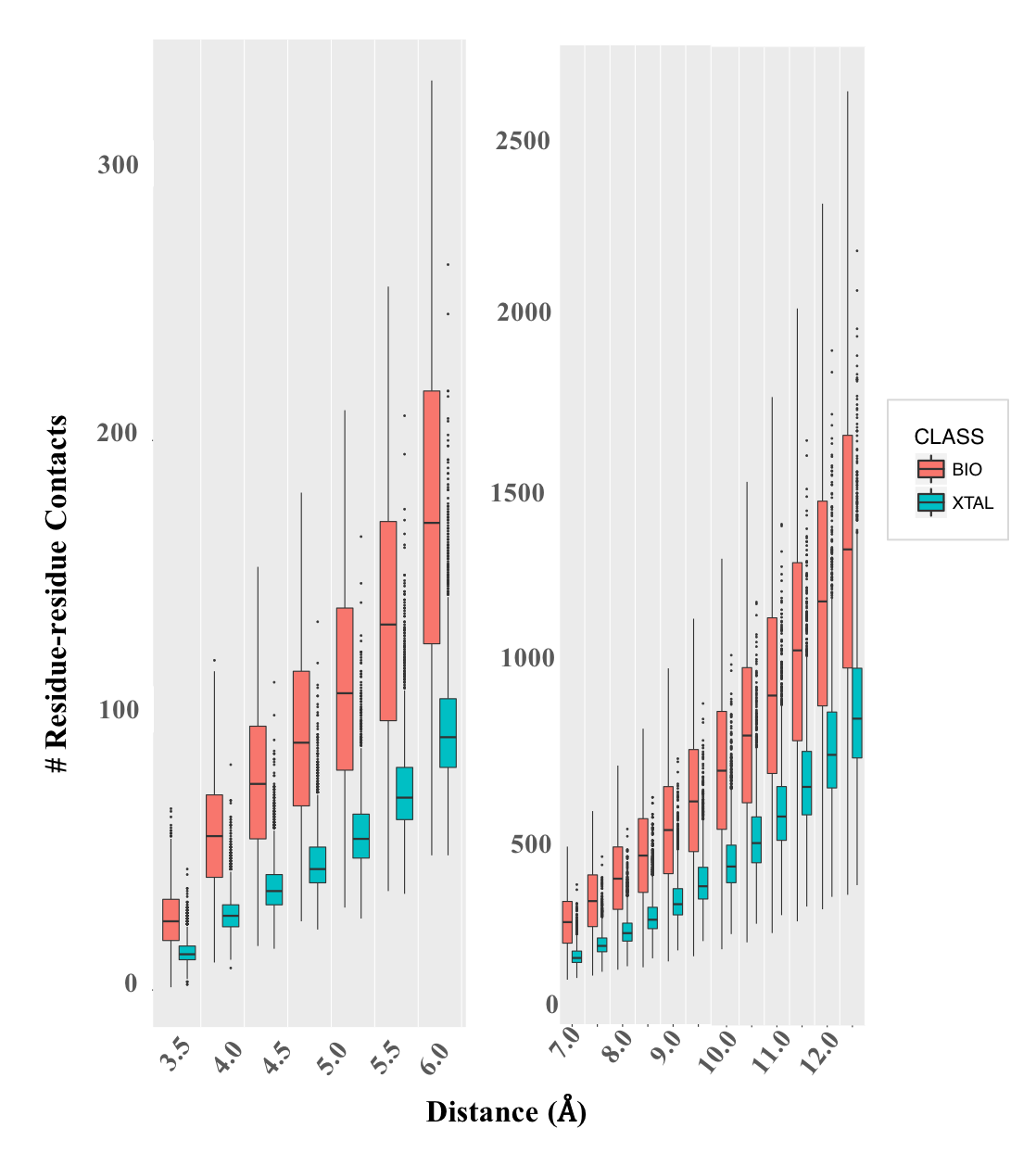

Supplement: Supplementary file 1 — Figure S1. Boxplot of the RCs as function of the distance cut-off. Table S1. Evaluation of machine learning accuracy models VS distance cut-off. Table S2. Feature selection on the final predictive model. (ZIP 207 kb) [file 12859_2018_2414_MOESM1_ESM.zip › S2-S1.png]
